# Asthma, COPD, Respiratory, and Allergic Health Effects in an Adult Population Living near an Italian Refinery: A Cross-Sectional Study

**DOI:** 10.3390/healthcare11071037

**Published:** 2023-04-04

**Authors:** Mariangela Valentina Puci, Ottavia Eleonora Ferraro, Maria Cristina Monti, Marco Gnesi, Paola Borrelli, Ennio Cadum, Pietro Perotti, Simona Migliazza, Simona Dalle Carbonare, Cristina Montomoli, Simona Villani

**Affiliations:** 1 Unit of Biostatistics and Clinical Epidemiology, Department of Public Health, Experimental and Forensic Medicine, University of Pavia, 27100 Pavia, Italy; 2 Laboratory of Biostatistics, Department of Medical, Oral and Biotechnological Sciences, University “G. d’Annunzio” Chieti-Pescara, 66100 Chieti, Italy; 3 Health Protection Agency of Pavia (ATS Pavia), 27100 Pavia, Italy

**Keywords:** asthma, respiratory disease, cross-sectional study

## Abstract

Background and aim. Asthma and chronic obstructive pulmonary disease (COPD) are leading causes of morbidity and mortality worldwide. Globally, 545 million people suffer from chronic respiratory diseases with a wide geographical variability. Risk factors for asthma are both genetic and related to several environmental factors (internal and external pollutants); these also have an important role in the occurrence of COPD. The aim of this study was to describe the prevalence of asthma, COPD, and asthma/COPD overlap (ACO) in an adult population living in two municipalities located in the Po Valley. Methods. A standardized questionnaire on respiratory symptoms and sociodemographic characteristics was self-administered to a random sample of the adult population aged 20–64 years, living near a refinery in Northern Italy during the period between 2016 and 2019. Logistic and multinomial regression were implemented to explore factors associated with asthma, COPD, and ACO. Results. In total, 1108 subjects filled out the questionnaire, the mean age was 48.02 ± 12.34 years (range 21–68), and 53% of the respondents/participants were female. Half of the responders were non-smokers, but the frequency of current and former smokers was significantly greater in men than in women (*p* < 0.001). The likelihood of being a probable case of asthma decreased with increasing age and increased for smokers. Tobacco smoke was associated with the presence of COPD and ACO. Conclusion. Respiratory diseases such as asthma and COPD are common in the general population, with differences among countries worldwide. Our findings show, on the basis of the main confirmed risk factor, namely smoking, that it is useful to plan target programs and actions in order to reduce smoking, thus improving the quality of life in public health.

## 1. Introduction

Respiratory diseases like asthma and chronic obstructive pulmonary disease (COPD) are chronic conditions that require an important contribution from patients, society, and the healthcare system [1]. Moreover, these diseases also play an important role in the patient’s perception of quality of life [2], although the assessment of well-being related to health is very subjective [3].

In 2019, asthma affected an estimated 262 million (m) people, with slight differences between males and females (127 m vs. 136 m, respectively), and caused 455,000 deaths with a wide geographical variability [4]. In Italy, a recent study by the Mild/Moderate Asthma Network (MANI) reported that asthma affected ~2 million people, with an estimated prevalence of ~4% [5]. In 2019, COPD affected 212 million people (105 m males and 107 m females), with a global prevalence of ~10% among people aged 30–79 years, with values that varied among different regions [6]. These numbers are rising [7,8] and represent a source of concern because these respiratory diseases have become an increasingly important cause of mortality and morbidity worldwide, not only in adults but also in children.

The risk factors for asthma are mainly genetic, but usually attributable to environmental factors; these also perform an important role in the occurrence of COPD, which is characterized by lung airflow limitation and tissue destruction, mainly due to exposure either to harmful substances or to vapor, gases, dust (especially biological), and fumes (VGDF) [9,10,11,12]. COPD is the third leading cause of mortality worldwide; despite the advancement in its management and treatment, mortality in these patients is still high [13].

Epidemiological studies and scientific literature showed that environmental changes represent one of the main causes of the increase in cases of asthma, suggesting that exposure to air pollution due to industrial facilities through volatile organic compounds (VOCs) [14], sulfur dioxide (SO_2_) [15] and hydrocarbons [16], motor vehicle emissions, and other environmental factors such as tobacco smoke are associated with this respiratory disease [17,18,19]. Air pollution is closely linked to some industrial activities, as evidenced by a recent Italian study showing an association between emissions from an oil refinery and reduction in lung function and airway inflammation [20]. As people spent 90% of their time indoors, it is important to take into account indoor pollution as well. Indoor air is typically a mixture of chemical and biological pollutants, e.g., indoor allergens, biological matter, mold, endotoxin, and nitrogen dioxide, which can be related to episodes of rhinitis and asthma exacerbation [21,22,23].

The prevalence of asthma, as well as of COPD, in epidemiological studies is heterogeneous, as the methods of diagnosis of probable disease are different. As for asthma, spirometry is the first test used to make a diagnosis, followed by bronchodilator reversibility testing; however, a task force of the European Respiratory Society systematically reviewed the existing literature on the diagnostic accuracy of the different tests used and provided recommendations for clinical practice [24]. Other ways to detect probable cases of asthma include self-reported diagnosis by self-reported respiratory symptoms, such as wheezing, usually through a screening questionnaire [25,26]. Therefore, because, currently, there is not a sole test for making an asthma diagnosis [27,28], its prevalence may even be underestimated, with wide variation among countries [29]. The diagnosis of COPD is made by a pulmonary function test called spirometry. The diagnostic steps may include the use of other diagnostic tests, especially on people with relevant symptoms or risk factors [30].

In addition, data from previous studies revealed that, in patients with clinical features of both respiratory diseases (overlap of asthma and COPD, defined as ACO), a higher frequency of respiratory symptoms, exacerbations, comorbidity, a poor quality of life, and an increase of health care utilization were observed compared with those with either asthma or COPD. Furthermore, unlike asthma and COPD, there is currently no consensus on a strict definition of ACO [31].

In the 1990s, an important study was carried out to estimate the prevalence of asthma and respiratory symptoms, the European Community Respiratory Health Survey (ECRHS) [32]. The protocol of ECRHS provided detailed instructions for the survey methodology, especially for the use of questionnaires to collect all data about respiratory symptoms, lung function measurements, home environment, smoking, allergy tests, bronchial responsiveness, occupational exposure, and other information. Nowadays, The ECRHS questionnaire is validated and is widely used in many surveillance initiatives and surveys [32,33,34,35].

The present study aimed to estimate the prevalence of asthma, COPD, and ACO within an adult population (20–64 years) living near an oil refinery located in the north of Italy (Lombardy Region, Province of Pavia). Our findings may be useful for local healthcare professionals, decision-makers, and stakeholders to improve, analyze, and evaluate the burden of these respiratory diseases, in order to implement and optimize clinical programs and public health strategies.

## 2. Material and Methods

### Study Design, Study Area and Population

An observational cross-sectional study was carried out within the CONSAL Project (Studio Conoscenza e Salute). This project aimed at describing the health status of subjects living in the province of Pavia (a city located in Lombardy region, northern Italy), in two small municipalities neighboring an oil refinery—the former is on the north-east border and the latter is on the north-west border, and they are about 2.5 km and 2 km away from the facility, respectively.

The province of Pavia is located in the Po Valley, one of the most polluted areas in Europe [36], as it has been estimated that there are emissions into the atmosphere of about 800,000 tons of air pollutants such as NO_x_, PM, and NH_3_ per year [37].

The oil refinery was built in 1963 and covers an area of about 320 hectares. From 2011 to 2016, it was enlarged with a new unit that should allow the almost total conversion of heavy fuels, maximizing the production of light distillates [38,39].

The target population was represented by people living in two municipalities located near the refinery. The inclusion criteria were to be residents in the two municipalities, according to the register of the Local Health Agency, as well as to be aged 20–64 years in 2016 and of both sexes. From this eligible population of 3654 people, 2500 subjects were randomly selected using a proportional stratified approach (width of municipalities: big/small = 2:1).

## 3. Outcomes, Other Variables and Data Collection

### 3.1. Outcomes: Case Definition of Asthma, COPD and ACO

A standardized self-administered short questionnaire derived from the European Community Respiratory Health II survey (ECRHS-II) and from the Gene Environment Interactions in Respiratory Diseases (GEIRD studies) [40,41] was used to collect information on respiratory health. The accuracy and reliability of the questionnaire contents were evaluated in a previous study [42].

Subjects were defined as having asthma (a probable case of asthma) if they had reported at least one of the following conditions in the screening questionnaire: having had an asthma attack within the past 12 months; taking asthma drugs (including sprays, aerosols, and tablets); having asthma; and having received a diagnosis of asthma by a physician [43,44]. Furthermore, we also defined as probable cases of COPD the subjects reporting in the questionnaire that they had a cough and phlegm almost every day for at least 3 months a year [45].

As a subject may be either not affected by any of the health outcomes, or affected by asthma alone, COPD alone, or by both conditions, this possibility was taken into account in the outcome of asthma and COPD (ACO), using the absence of respiratory diseases as the reference category in the model [46].

### 3.2. Other Variables

The same questionnaire was used to gather information on respiratory outcomes and on factors of interest such as sociodemographic characteristics (age and sex), smoking habits (defined as non-smokers, smokers, and former smokers), and asthma treatment. Moreover, respiratory symptoms (occurred in the past year) were described as follows: presence of wheezing, hay fever/allergic colds, cough, and phlegm. Additionally, information about hospitalizations due to respiratory conditions was collected.

### 3.3. Data Collection Framework

This study was carried out between 2016 and 2019 because more “waves” were needed to enroll people from the two municipalities.

## 4. Sample Size

A sample size of 2500 subjects was required for this study. This result was obtained considering a prevalence of asthma equal to 6% (Italian reference value), a 95% confidence level, a 0.6% margin of error, and a possible response rate of 80%.

## 5. Statistical Analysis

Sample characteristics were described using summary statistics as follows: mean and standard deviation (sd) for the quantitative variables and absolute and relative frequencies (expressed in percentages as well) for the qualitative variables. The prevalence of asthma and COPD in the study population was calculated as the percentage of subjects having well-defined respiratory disease symptoms based on questionnaire answers. Differences in the distribution of respiratory symptoms or conditions by the presence of respiratory diseases were analyzed using Pearson Chi square test. First, we implemented a logistic regression to evaluate which factors were associated with asthma alone and COPD alone. In this analysis, the results were reported as odds ratio (OR) with their 95% confidence intervals (95% CI). Secondly, a multinomial logistic regression was employed to explore factors associated with ACO, because, in this case, there is no natural ordering among categories of this outcome. In the multinomial model, the disease-free group was chosen as the baseline category. In this analysis, the measure reported was relative risk ratios (RRRs) with 95% confidence intervals. Finally, in order to test the goodness-of-fit of both models (logistic and multinomial regression), the Hosmer-Lemeshow test was implemented. A two-tailed *p*-value of less than 0.05 was considered as the statistical significance level. All analyses were conducted using STATA/SE statistical software for Windows, version 17 [47]. 

## 6. Results

Out of the 2500 participants enrolled, 1108 answered the questionnaire: 833 (75%) subjects were residents in the bigger municipality and 275 (25%) in the smaller one, with a global response rate of 50%. The mean age of study participants was 48 (±12.3) years (range 21–68), with a slightly greater proportion of females (53%), as reported in Table 1. Five percent of responders were 68 years old at the end of the enrollment. 

Regarding smoking habits, about half of the respondents were non-smokers (49%), followed by former and current smokers, who were equally distributed (26%, Table 1). The former smokers had quit smoking on average 17.0 ± 11.8 years before (median and interquartile range 16; 6–27 years before). The frequency of current and former smokers in our study was significantly greater in males than in females (62% vs. 41%, *p* < 0.001).

## 7. Respiratory Outcomes 

Regarding the study outcomes, 24 subjects did not provide any details and information on asthma and/or COPD in their questionnaire, and they were subsequently excluded from the analysis.

The overall prevalence of probable asthma was 11% (Table 1), with no differences between females (11.3%) and males (10.6%) (*p* = 0.715). The overall prevalence of probable COPD was 25%, with a significantly higher occurrence in males (29.0%) compared with females (21.6%) (*p* = 0.005). Similarly, as seen for asthma, the prevalence of ACO in males (6.2%), although slightly higher, overlaps with that in females (5.6%) (*p* = 0.702). 

### 7.1. Respiratory Symptoms Related to Outcomes

Table 2 shows the distribution of different symptoms stratified by asthma, COPD, ACO subgroups, and for the entire sample.

Among the 1067 participants who responded to questions about respiratory symptoms, wheezing occurred in 11%, asthma attack in 5%, hay fever and allergic colds in 18%, cough in 20%, and phlegm in 18%. Moreover, 4% of participants declared the use of anti-asthmatic drugs and 6% reported hospitalizations for respiratory conditions.

Comparison among different respiratory conditions revealed that the wheezing proportion was higher in subjects only affected by asthma (26%) than in those only affected by COPD (17%); this proportion significantly increased if both conditions were present (58%; *p* < 0.001). Hay fever and allergic colds were significantly higher in the asthma subgroup compared with the COPD and ACO subgroups (57%, 24%, and 48% respectively; *p* < 0.001). Cough and phlegm were not present among the asthmatic subjects, while they were prevalent in the other two subgroups. Furthermore, the use of anti-asthmatic drugs differed between the asthma and ACO subgroups, with proportions ranging from 26% to 52% (*p* <0.001). 

Finally, 17% of asthmatic and 9% of COPD subjects declared that they had been hospitalized for respiratory conditions. As reported in Table 2, this proportion significantly increased if both respiratory disease conditions were present (29% ACO subgroup; *p* < 0.001).

### 7.2. Factors Associated with Respiratory Outcomes

Table 3 and Table 4 show the results of the logistic regression analyses assuming probable asthma and probable COPD cases as model outcomes (i.e., dependent variables). These models showed that the factors associated with a probable case of asthma were smoking habits (smokers vs. non-smokers: OR (95%CI): 2.105 (1.335–3.317)) and age (OR (95%CI): 0.976 (0.960–0.991)), whereas for COPD, the associated factors were sex (females vs. males OR (95%CI): 0.744 (0.556–1.000)) and smoking habits (smokers vs. non-smokers: OR (95%CI): 2.595 (1.855–3.630)). 

## 8. ACO 

The multinomial logistic regression analysis included sex, smoking habits, and age as independent variables (Table 5). The results from the model showed that an increase in age of 1 year significantly reduced the relative risk of asthma by 3%. The category associated with both COPD and ACO diseases was being a current smoker, adjusting for other covariates. The relative risk of having COPD was more than double in smokers (RRR = 2.440, 95%CI: 1.669–3.569) compared with non-smokers. Furthermore, in smokers, the relative risk of having both asthma and COPD was three times higher (RRR = 3.649, 95%CI: 1.969–6.763) than in non-smokers. In addition, being a female reduced the relative risk of having COPD by 32% (RRR = 0.680, 95%CI: 0.490–0.945). The Hosmer-Lemeshow goodness-of-fit test indicated that the model fits the data well (χ^2^ = 16.173, *p* = 0.882).

## 9. Discussion

In the present study, based on data collected from the CONSAL project, we have reported up-to-date information on the prevalence of asthma, COPD, and ACO among an adult population living near an oil refinery in Northern Italy using a validated questionnaire. Together with this estimation, data about the distribution of respiratory symptoms and risk factors associated with the study outcomes were reported.

The main findings of this study are as follows:

(1) The likelihood of being a probable case of asthma decreased with increasing age and increased for smokers;
(2) The likelihood of probable cases of COPD was associated with sex (higher COPD prevalence among males) and smoking habits (higher risk among smokers compared with non-smokers);
(3) Smokers compared with non-smokers had a higher risk of having both respiratory conditions;
(4) Distributions of respiratory symptoms were higher when asthma and COPD coexisted (ACO).

The first three findings were supported by the multinomial logistic regression model, where significant associations were found between age, sex, smoking, and respiratory outcomes.

It is known that asthma and COPD, being long-term conditions, affect not only children but also adults, and these diseases are public health issues that occur in both high- and low-income countries [48,49]. Starting from this point, the prevalence of asthma (around 11%) found in our study was similar to that detected in a town near an oil refinery in Jordan [50]—in the literature, other specific findings on asthma prevalence basically refer to children [51,52] or a selected population such as refinery workers [53]. Moreover, this finding regarding asthma is higher than that within the Italian general population, as highlighted in a world health survey, in the MANI study, and in the Italian National Institute of Statistics (ISTAT) report (6.26%, 4%, and 6%, respectively) [5,54,55]. This difference could be due to the selected age sample in our study (20–64 years) in comparison with the results found in samples from all ages. With regard to age, the literature data showed similar results for both the pediatric and adult population, where an increase in age was significantly associated with a reduced probability of having asthma and its symptoms [56,57]. In addition, regarding smoking, our results are similar to other studies where smoking was not associated with the prevalence of asthma [57,58].

On the other hand, our findings confirm that smoking habits have a relevant role in the probable cases of COPD or in the overlap of diseases, as reported in the literature [59]. More precisely, in our study, the probability of smokers of being affected by these respiratory problems was double (for COPD) and triple (for ACO), consistent with what was reported by the WHO and other studies [46,48,60]. Regarding sex, in our study, females had a decrease in the prevalence of probable COPD, likely owing to the lower percentage of current and former smokers (41% vs. 62% in males *p* < 0.001). The lower prevalence of COPD in females than in males is in line with that found in a recent meta-analysis, where COPD prevalence was higher in males for both the overall and age-specific sample (overall 9.23% vs. 6.16%; age > 40 years 11.55% vs. 7.47%, respectively) [61]. Additionally, the prevalence of COPD in our sample was higher than in the European and north-eastern Italian general population [62].

Regarding ACO, a recent meta-analysis (2019) found that its prevalence ranged from 0.3% to 5% in the general population [31], but even in this case, our finding is around 6% higher. Moreover, once again, consistent with this meta-analysis, our data highlighted that the presence of respiratory symptoms increases when both conditions occur (ACO). More precisely, this result clearly emerged in the case of wheezing, asthma attack, cough, phlegm, and self-reported hospitalization for respiratory conditions, because their frequency was higher in the ACO subgroup than in the single disease (asthma or COPD) subgroups.

Finally, the discrepancies in asthma and COPD prevalence discovered in the present study with the evidence from the other studies might be attributed to different factors related to the lack of standardized protocols/diagnosis, genetic susceptibilities of subjects, or diverse environmental exposures among different geographical areas.

This study has several limitations. The first is the recall bias, as the possible diagnoses of asthma and COPD were based on answers of a self-reported questionnaire, not on diagnostic tests or medical records. In any case, ECRHS studies have proved a reasonable reliability of the definitions provided in the Section 2 [40]. Overestimation of the results may also be due to the percentage obtained in the response rate, even if this value was consistent with other study populations [46]. Furthermore, the lack of a confirmation from spirometry testing could also bring different results [63]. In conclusion, the cross-sectional design does not allow to infer causality. For this reason, longitudinal studies are needed to clarify the relationship between respiratory conditions and other variables, among which are external and internal pollutants.

## 10. Conclusions

This study provides recent estimates of the prevalence of asthma and COPD in the population of two municipalities close to an Italian oil refinery.

The present findings show that the prevalence of asthma increased compared with the general Italian population, but it is similar to that reported in a study conducted near a refinery. A similar increase is observed for COPD compared with other studies carried out on the general population.

On the basis of the major risk factors confirmed in this study, namely smoking, the findings may be useful in health policies for decision-makers to plan programs and actions to reduce tobacco smoke and, consequently, smoking-related respiratory diseases, or to identify possible clusters of subjects at risk, e.g., starting with their ages.

In the future, it would also be interesting to collect ad hoc data to assess how these respiratory diseases can interact with other diseases to plan different types of health interventions in public health.

Finally, it will be important to understand and quantify the impact of asthma and COPD on the quality of life of adults, as well as which strategies could be adopted to improve children’s health. 

Further research is recommended, including studies on other populations such as children or the elderly, as well as longitudinal studies focused on occupational exposure, levels of contaminants or air pollutants, and clinical outcomes in order to better evaluate the impact of oil refineries on the health of the population.

## Figures and Tables

**Table 1:** Profile of respondents.

	Respondents	Ratio (M/F)
Age (years), mean (sd)	48 (12.34)	
Sex, %(n)		
Male	47% (520)	
Female	53% (588)	
Smoking, %(n)		
Non-smokers	49% (532)	195/377
Former smokers	26% (281)	162/119
Smokers	26% (282)	159/123
Respiratory outcomes		
Probable case of asthma, %(n)	11% (119)	54/65
Probable case of COPD, %(n)	25% (273)	148/125
Probable case of asthma and COPD overlap (ACO), %(n)	5.9% (65)	32/33

**Table 2:** Distribution of respiratory symptoms or conditions by the presence of respiratory diseases.

	Overall	Disease-Free	Asthma Alone	COPD Alone	ACO	*p*-Value *
	N = 1067	N = 746	N = 54	N = 202	N = 65	
Wheezing	11% (112)	3% (25)	26% (14)	17% (35)	58% (38)	<0.001
Asthma attack	5% (52)	0% (0)	24% (13)	0% (0)	60% (39)	<0.001
Anti-asthmatic drugs	4% (47)	0% (0)	26% (14)	0% (0)	52% (33)	<0.001
Hay fever/allergic colds	18% (192)	11% (82)	57% (31)	24% (48)	48% (31)	<0.001
Cough	20% (213)	0% (0)	0% (0)	78% (155)	89% (58)	<0.001
Phlegm	18% (193)	0% (0)	0% (0)	70% (140)	84% (53)	<0.001
Hospitalizations for respiratory conditions	6% (60)	2% (14)	17% (9)	9% (18)	29% (19)	<0.001

Results are shown without missing data. Data are expressed as percentages and frequencies (n), * Pearson Chi square test.

**Table 3:** Variables associated with probable cases of asthma: logistic regression analysis.

Probable Asthma Cases Yes vs. No	ORs [95%CI]	
Sex		
Female vs. male	1.194 [0.799–1.785]	
Smoking		
Former smokers vs. non-smokers	1.271 [0.749–2.158]	
Smokers vs. non-smokers	2.105 [1.335–3.317]	**
Age (years)	0.976 [0.960–0.991]	**

** *p* < 0.010.

**Table 4:** Variables associated with probable cases of COPD: logistic regression analysis.

Probable COPD Cases Yes vs. No	ORs [95%CI]	
Sex		
Female vs. male	0.744 [0.556–1.000]	*
Smoking		
Former smokers vs. non-smokers	1.185 [0.814–1.725]	
Smokers vs. non-smokers	2.595 [1.855–3.630]	**
Age (years)	1.000 [0.988–1.011]	

* *p* < 0.050, ** *p* < 0.010.

**Table 5:** Variables associated with asthma, COPD, and both: multinomial logistic regression analysis (n = 1059).

Probable Asthma Cases vs. Disease-Free Group (n = 54)	RRRs [95%CI]
Sex	
Female vs. male	1.184 [0.665–2.111]
Smoking	
Former smokers vs. non-smokers	1.113 [0.524–2.362]
Smokers vs. non-smokers	1.736 [0.895–3.370]
Age (years)	0.965 [0.944–0.987] **
**Probable COPD Cases vs. Disease-Free Group** **(n = 202)**	
Sex	
Female vs. male	0.680 [0.490–0.945] *
Smoking	
Former smokers vs. non-smokers	1.141 [0.749–1.739]
Smokers vs. non-smokers	2.440 [1.669–3.569] **
Age (years)	1.001 [0.987–1.015]
**ACO Cases vs. Disease-Free Group (n = 65)**	
Sex	
Female vs. male	1.022 [0.598–1.748]
Smoking	
Former smokers vs. non-smokers	1.554 [0.751–3.213]
Smokers vs. non-smokers	3.649 [1.969–6.763] **
Age (years)	0.986 [0.964–1.007]

* *p* < 0.05, ** *p* < 0.01. The disease-free group is made of subjects with no respiratory outcomes (asthma, COPD, or both).

## Data Availability

The data presented in this study are not publicly available due to privacy restrictions.
